# Antinociceptive and anti-tussive activities of the ethanol extract of the flowers of *Meconopsis punicea* Maxim

**DOI:** 10.1186/s12906-015-0671-y

**Published:** 2015-05-22

**Authors:** Xiaofei Shang, Dongsheng Wang, Xiaolou Miao, Yu Wang, Jiyu Zhang, Xuezhi Wang, Yu Zhang, Hu Pan

**Affiliations:** Key Laboratory of New Animal Drug Project, Gansu Province, Key Laboratory of Veterinary Pharmaceutical Development of Ministry of Agriculture, Lanzhou Institute of Husbandry and Pharmaceutical Sciences of Chinese Academy of Agricultural Science, Lanzhou, 730050 People’s Republic of China; Department of Emergency, Lanzhou General Hospital of PLA, Lanzhou, 730050 People’s Republic of China

**Keywords:** *Meconopsis punicea* extract, Antinociceptive activity, Formalin test, Antitussive activity

## Abstract

**Background:**

As an important traditional Tibetan (veterinary) medicine, the flowers of *Meconopsis punicea* (family *Papaveraceae*) have been used to treat pain, fever, cough, inflammation, liver heat and lung heat of humans and animals by local people for thousands of years. In this paper, we aimed to investigate the antinociceptive and anti-tussive activities of the ethanol extract of *M. punicea* (EEM).

**Methods:**

Firstly, HPLC was used to analyze the main constituents of the ethanol extract of *M. punicea*. In animal experiments, the acetic acid-induced writhing response test, hot plate test, barbiturate-induced sleeping time and formalin tests were used to evaluate the antinociceptive activity. Then, ammonia-induced coughing and sulfur dioxide-induced coughing tests in mice as well as the phenol red secretion in trachea test were used to investigate the anti-tussive activity of the extract. Finally, an acute toxicity study was carried out.

**Results:**

The results showed that alkaloids and flavonoids were the main compounds in the ethanol extract of *M. punicea* flowers. The extract at 125, 250 and 500 mg/kg had good antinociceptive and anti-tussive activities in mice with a dose-dependent manner.

**Conclusions:**

These findings suggested that EEM has significant bioactivities, and the active components of *M. punicea* should be studied further.

## Background

**Fig. 1:**
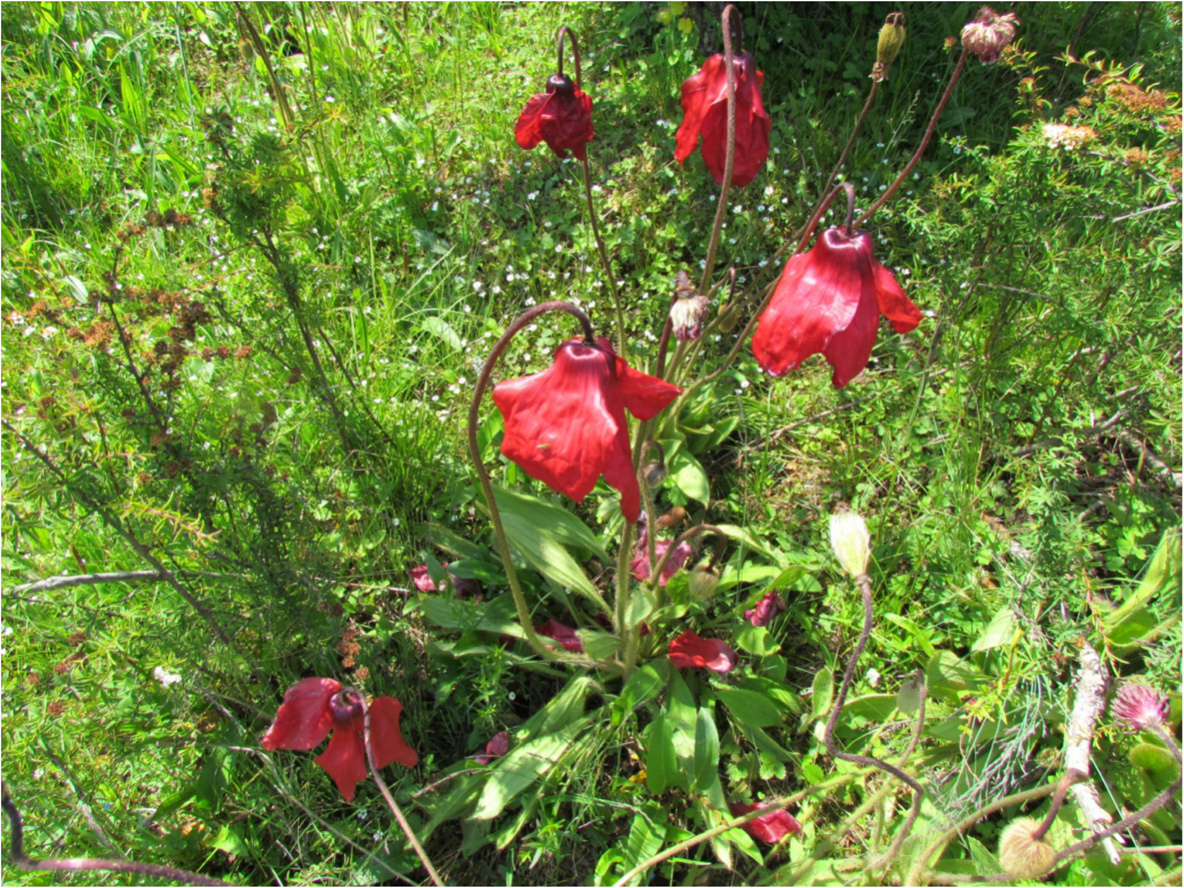
Picture of *Meconopsis punicea* Maxim

*Meconopsis punicea* Maxim. (Hong Hua Lv Rong Hao) is a perennial herb (family *Papaveraceae*) that grows from 30 cm to 70 cm tall. In China, as a traditional Tibetan (veterinary) medicine, it grows in alpine scrub and alpine meadows with shady and half-shady slopes at altitudes of 3000–4800 m and is distributed in the northeastern part of Tibet, southeastern part of Qinghai, western part of Sichuan and southern part of Gansu provinces (Fig. 1). The flower of *M. punicea* has been used to treat pain, fever, cough, inflammation, liver heat and lung heat of humans and animals by local people for thousands of years, and about 5 preparations containing *M. punicea* with other medicines have been listed in ’Drug Standard of Ministry of Public Health of the People’s Republic of China (Tibetan medicine volume)’. Meanwhile, the beautiful flowers are also used as ornamental plants in the Tibetan region [1].

Recently, the chemical composition of *M. punicea* was studied. And as the main components of the aerial part, a serial of alkaloids were identified, such as karachine, valachine, (−)-mecambridine, berberine, protopine and alborine. Meanwhile, flavonoids, including luteolin, apigenin, hydnocarpin and isorhamnetin, were also isolated and reported along with canillic acid, cinnamic acid and other components [2–4]. But up to now, no one has used modern technology to study its pharmacological effects.

In our field investigation of folk veterinary medicine, *M. punicea,* which has used to treat pulmonary disease, pain and inflammation by folk veterinarians for a long period of time in some regions of the Sichuan and Gansu provinces, was identified [5]. In this paper, considering *M. quintuplinervia* and relative species of the genus have been reported to exhibit analgesic and sedative activities [6, 7], and the traditional uses of *M. punicea* in alleviating pain and reducing cough and inflammation [5], we systematically evaluated the analgesic, anti-tussive and expectorant activities of the extract of *M. punicea* flowers.

## Methods

### Plant material

The herbal sample of *M. punicea* (34.02 ^o^ N, 102.74° E, 3531 m altitude) was collected at Rierlang Mountain of Ruoergai County in Sichuan province, China in July of 2011. The raw material was identified by Prof. Zhigang Ma, Pharmacy College of Lanzhou University, China. A voucher specimen with accession number ZSY028 was submitted to the Herbarium of Traditional Chinese Veterinary Medicine (Lanzhou Institute of Husbandry and Pharmaceutical Sciences of CAAS, China).

### Extraction

The ethanol extract of *M. punicea* (EEM) was prepared as follows. Briefly, 45 g of *M. punicea* flowers was refluxed in 80 °C with 500 ml of 95 % ethanol 3 times, and each reflux time was 2 h. After filtration, the solvent was evaporated in a rotary evaporator. The extract was vacuum dried at 60 °C. The yield was 14.27 %.

### Drugs

Acetic acid (Tianjing Chemical Reagent Company, Tianjing, China); aspirin (Hefei Jiulian Pharmaceutical Company, Anhui, China); morphine (The First Pharmaceutical Company of Shenyang, Shenyang, China); phenol red (The Third Chemical Reagent Factory of Shanghai, Shanghai, China). The standard compounds of berberine, luteolin and apigenin (purity ≥ 98 %) were obtained from Shanghai R&D Centre for Standardisation of Chinese Medicines (China).

### Animals

Male or female Balb/C mice (20 ± 2 g) were obtained from the Department of Animal Center, Lanzhou Institute of Biologicals (Lanzhou, China). They were kept in plastic cages at 22 ± 2 °C with free access to pellet food and water. This study was carried out in accordance with the Regulation for the Administration of Affairs Concerning Experimental Animals (State Council of China, 1988), and was approved by the Ethics Committee of Lanzhou Institute of Husbandry and Pharmaceutical Sciences of Chinese Academy of Agricultural Science (Lanzhou, China).

### Phytochemical screening

The ethanol extract of *M. punicea* (EEM) was qualitatively tested for the detection of carbohydrates, saponins, flavonoids, tannins, alkaloids, glucosides and steroids following standard procedures [8].

RP-HPLC analysis of EEM was performed on a Waters apparatus (two solvent delivery systems, model 600, and a Photodiode Array detector, model 996), using a gradient solvent system comprised of formic acid in water (pH 3.0) (A) and CH_3_CN (B). The gradient profile was as follows: 0–20 min (90–85 % A), 20–35 min (85–70 % A), 35–70 min (70–40 % A) and 70–85 min (40–10 % A), at 0.5 ml/min. On-line UV spectra were recorded from 200 to 400 nm. Data acquisition and quantification were performed using Millenium 2.10 version software (Waters). A Sunfire C-18 column (250 mm*4.6 mm, 5 μm, Waters, Ireland) was maintained at ambient temperature (30.0 °C). The mobile phase was filtered through a Millipore 0.45 mm filter and degassed prior to use. The peaks were detected, and berberine, luteolin and apigenin were detected by comparison with chemical standards, which were identified with MS, ^1^H NMR and ^13^C NMR.

### Evaluation of antinociceptive activity of the ethanol extract

#### Acetic acid-induced writhing response

The test was carried out according to a previously described method [9]. Mice were randomly divided into five groups with ten animals in each group, namely, the normal control group, reference group, and three groups of EEM. The control group received normal saline (10 ml/kg, i.p.), and the reference group received aspirin (100 mg/kg, i.p.). EEM was intraperitoneally injected into each mouse at doses of 125, 250 and 500 mg/kg. After 30 min treatment, 0.7 % acetic acid (0.1 ml/10 g body weight) was administered intraperitoneally to each mouse. The mice were observed, and the number of abdominal constrictions and stretchings in a period of 0–30 min was counted.

### Hot plate test

The test was carried out according to a previously described method [10, 11]. EEM (125, 250, 500 mg/kg) was administered by intraperitoneal injection. The control group received normal saline (10 ml/kg, i.p.), and the reference group received morphine sulfate (5 mg/kg, i.v.). After 30 and 65 min, mice were individually placed on a heated plate at 55 ± 1 °C. The latency time of forepaw licking or jumping was determined.

### Barbiturate-induced sleeping time

The test was carried out according to a previously described method [12, 13]. The control group received normal saline (10 ml/kg i.p.), and the reference group received diazepam (12.5 mg/kg). EEM (125, 250, 500 mg/kg) was administered to the animals by intraperitoneal injection. After 30 min, sleep was induced by the intraperitoneal administration of 40 mg/kg pentobarbital. The latency time to sleep (time to lose the righting reflex) and sleeping time (duration of loss of the righting reflex) were measured.

### Formalin test

The test was carried out according to a previously described method [5, 14]. After treatment for 30 min with normal saline, EEM and positive drugs, 20 μl of 5.0 % formalin in normal saline was injected subcutaneously into a hind paw of each mouse. The time of lickings, stampings, and scratchings the injected paw were recorded and separated into the early phase (0–5 min) and late phase (15–40 min) after formalin injection. To elucidate the possible mechanism of action, morphine sulfate (5 mg/kg, i.v.) and aspirin (100 mg/kg, i.p.) were used as positive controls.

### Evaluation of anti-tussive activity of the ethanol extract

#### Ammonia-induced coughing in mice

The test was carried out according to a previously described method [15, 16]. Mice were randomly divided into five groups (n = 10). Thirty minutes after injecting the normal saline, EEM and codeine phosphate, each mouse was placed in a 1000 ml special glass chamber and exposed to 0.3 ml 25 % NH_4_OH produced by a nebulizer for 45 s. Then, mice were taken out and put in an open field. The cough frequency and latent period of coughing were recorded for 5 min, and the anti-tussive activity was assessed as the percentage of inhibition of the number of coughs.

### Sulfur dioxide-induced coughing in mice

The test was carried out according to a previously described method [15, 17], and mice were randomly divided into five groups. After treatment for 30 min with normal saline, EEM and codeine phosphate, a burette containing 2 ml of 50 % sulfuric acid solution was fixed to a flask containing 2 g of anhydrous sodium sulfite, and the acid was added to this sulfite to generate sulfur dioxide gas. Meanwhile, each mouse was placed in a 1000 ml special glass chamber, and the number of coughs was recorded for 3 min. The anti-tussive activity was assessed as the percentage of inhibition of the number of coughs.

### Phenol red secretion in mouse tracheas

The test was carried out according to a previously described method [15, 18], and mice were randomly divided into five groups. After treatment for 30 min with normal saline, EEM and NH_4_Cl, each mouse was treated with phenol red solution (5 % in saline solution, w/v, and 0.2 ml/20 g body weight). Thirty minutes after the application of phenol red solution, mice were sacrificed by cervical dislocation without damaging the tracheas. The trachea was dissected away from adjacent organs, and 2 ml of normal saline was used to wash 3 times. After ultrasonication for 15 min, 1 ml of a 5 % NaHCO_3_ solution was added to the normal saline, and the optical density was measured at 560 nm using LabTech UV-BlueStar Plus (Beijing LabTech. Inc. China).

### Acute toxicity study

The up-and-down or staircase method for acute toxicity testing was carried out as previously described [19]. 500 to 2000 mg/kg EEM was administered to mice through the intraperitoneal route with a gradual increase in dose. The behavioral changes and mortality of animals were observed continuously for the first 4 h and 7 days after the drug administration.

### Statistical analysis

The data obtained were analyzed using SPSS software program version 18.0 and expressed as the mean ± S.E.M. Data were analyzed by a one-way ANOVA followed by Student’s two-tailed *t*-test for the comparison between test and control and Dunnett’s test when the data involved three or more groups. P-values less than 0.05 (*P < 0.05*) were accepted as significant.

## Results

### Phytochemical screening

**Fig. 2:**
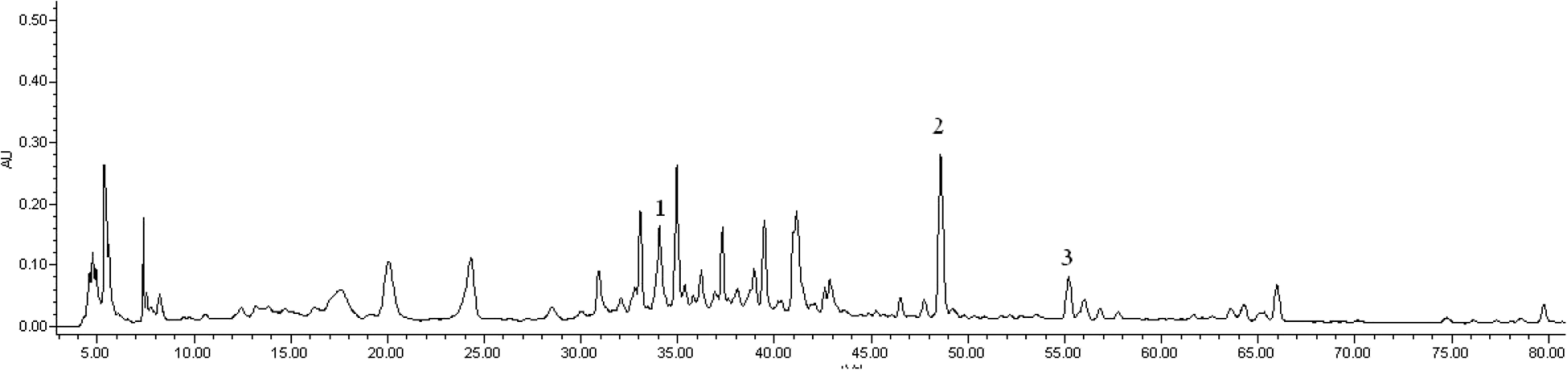
HPLC-DAD chromatogram of the ethanol extract of *M. punicea*. (1. Berberine, 2. Luteolin, 3. Apigenin)

After the preliminary phytochemical analysis, we found that alkaloids and flavonoids were the main constituents of EEM. In the HPLC analysis, berberine (1), luteolin (2) and apigenin (3) were found in EEM (Fig. 2).

### Acetic acid-induced writhing response

**Fig. 3:**
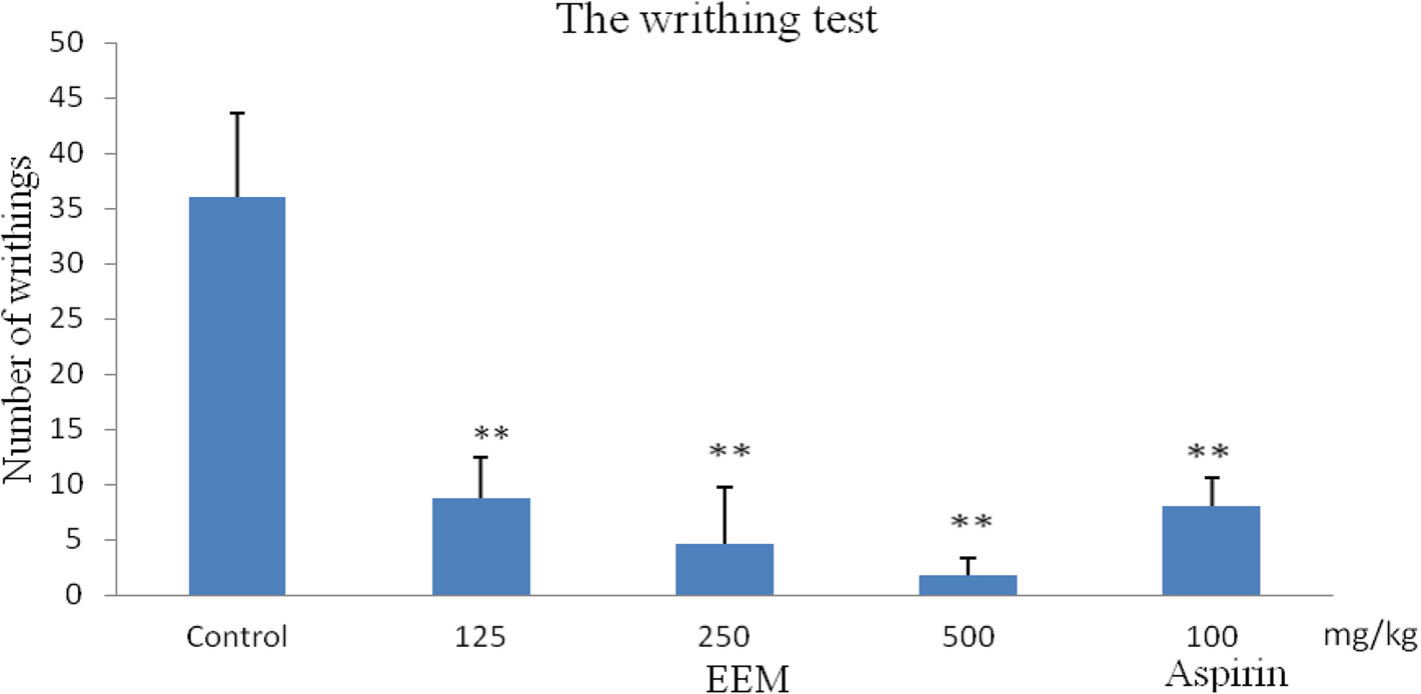
Antinociceptive activity of the ethanol extract of *M. punicea* in the writhing test. (**P < 0.01 compared with control.)

The results showed that EEM (500, 250 and 125 mg/kg) significantly restrained the writhing response induced by 0.7 % acetic acid with an inhibition rate of 95.28 %, 87.22 % and 75.56 % (*P < 0.01*) in a dose-dependent manner. As the positive drug, aspirin (100 mg/kg, i.p.) produced a 77.78 % reduction compared to the control. The results are shown in Fig. 3.

### Hot plate test

**Fig. 4:**
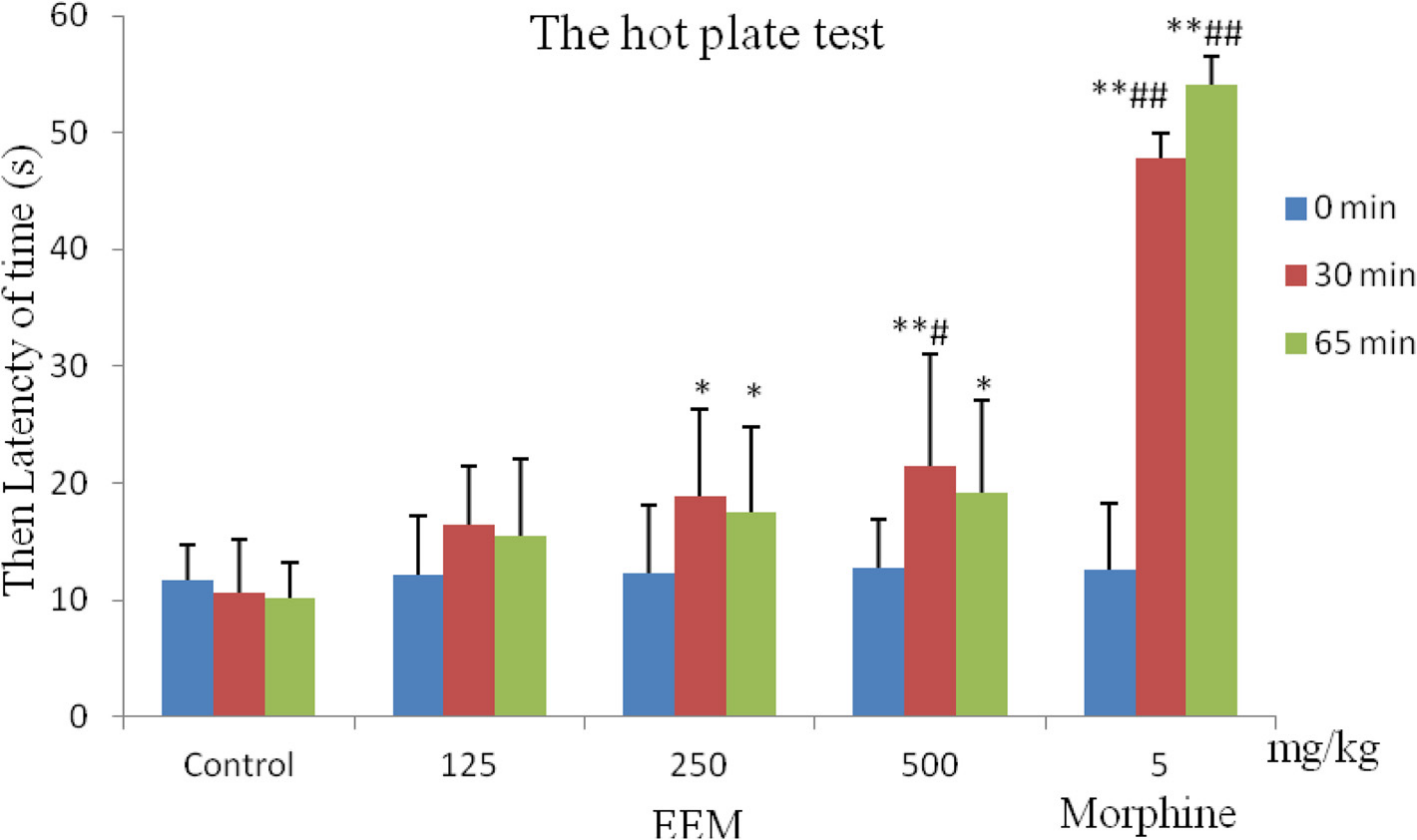
Antinociceptive activity of the ethanol extract of *M. punicea* in the hot plate test. (**P < 0.05, **P < 0.01 compared with latency time in the control group at each corresponding time. ^##^ *P* < 0.05, ^##^ *P* < 0.01 compared with the latency time at 0 min in each corresponding group.)

As shown in Fig. 4, EEM had antinociceptive activity in the hot plate test. Compared to the control group at 30 and 65 min, EEM (500 mg/kg) could prolong the latency time of mice (*P < 0.01*). At the same time, it also prolonged the latency time at 0 min in this group. Morphine sulfate (5 mg/kg, i.v.) markedly increased the pain threshold of mice at 30 min or 65 min after treatment (*P < 0.01*).

### Barbiturate-induced sleeping time

**Fig. 5:**
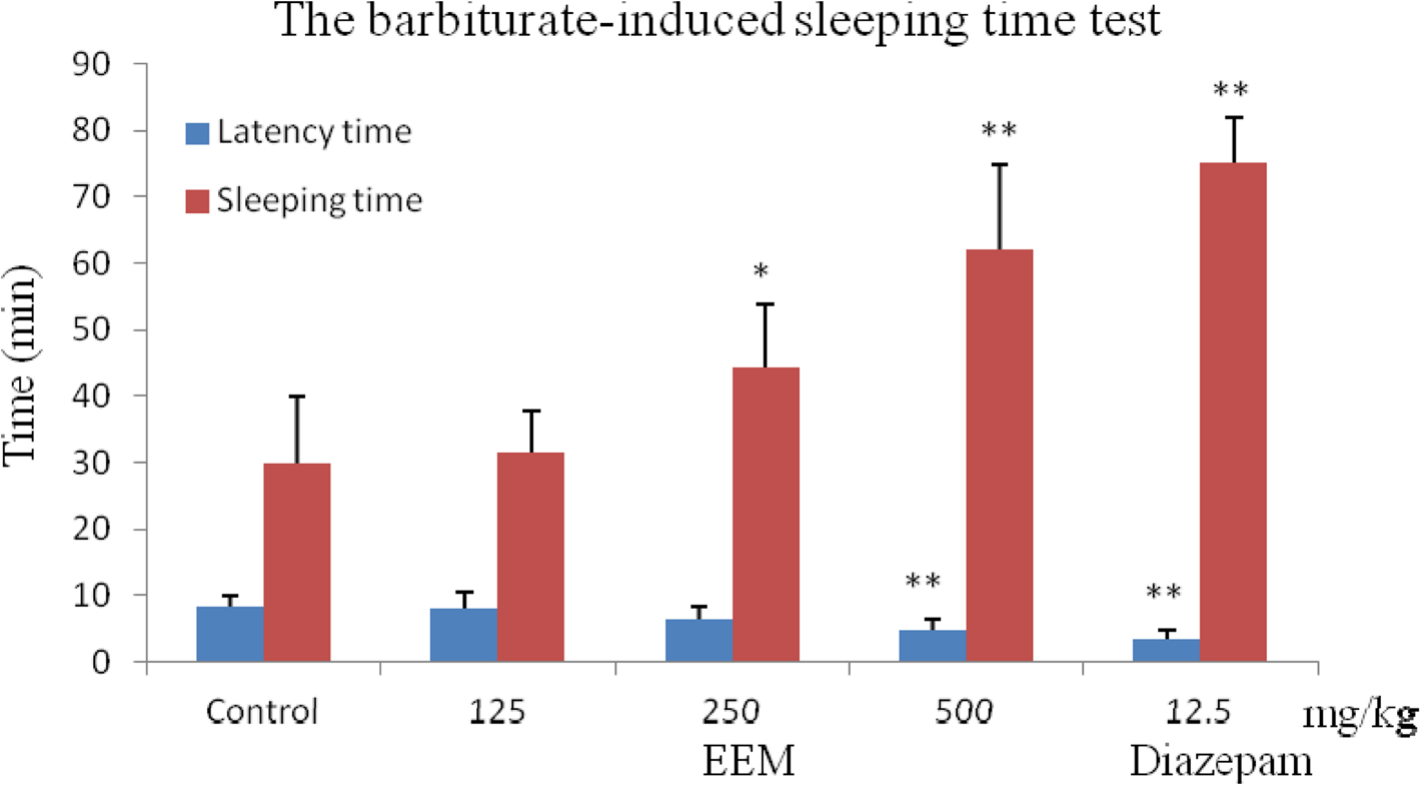
Antinociceptive activity of the ethanol extract of *M. punicea* in the barbiturate-induced sleeping time test. (**P < 0.05, **P < 0.01 compared with latency time in the control group at each corresponding time.)

On the basis of the above tests, we carried out the barbiturate-induced sleeping time test. The results showed that at 500 mg/kg, EEM significantly decreased the latency time of sleep and prolonged sleeping time of mice compared to the control group, and the latency time and sleeping time of mice were 4.9 and 62.1 min (*P < 0.01*), respectively. For the diazepam positive control, the latency time and sleeping time were 3.4 and 75.3 min (*P < 0.01*), respectively (Fig. 5).

### Formalin test

**Fig. 6:**
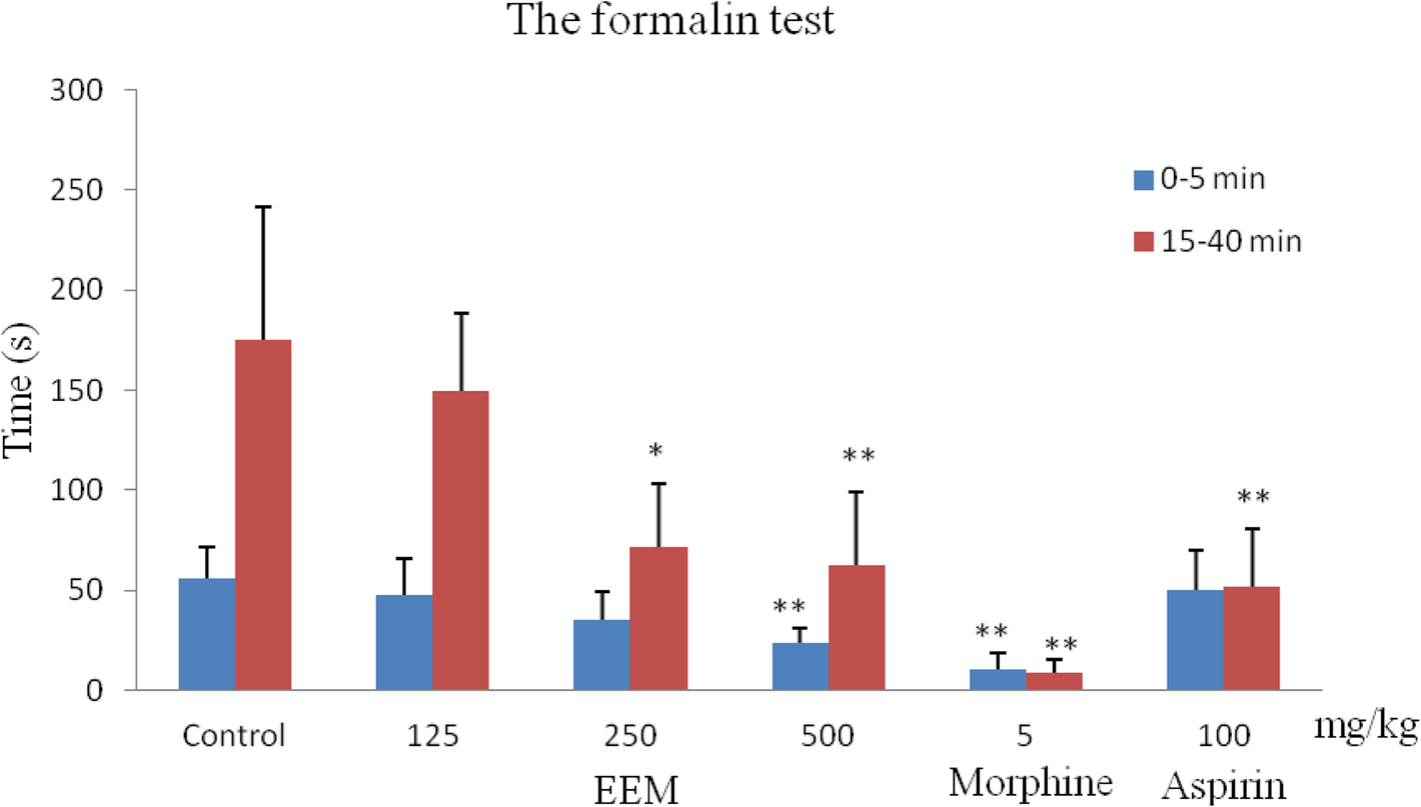
Antinociceptive activity of the ethanol extract of *M. punicea* in the formalin test. (**P < 0.05, **P < 0.01 compared with latency time in the control group at each corresponding time

As shown in Fig. 6, EEM had antinociceptive activity in the formalin test. In the early (0–5 min) and late phases (15–40 min), EEM could decrease the time of licking, stamping, and scratching induced by formalin in a dose-dependent manner, especially at 500 mg/kg, with an inhibition of 58.03 % and 64.19 % (*P < 0.01*). Morphine, used as the positive control, produced a marked reduction of 81.91 % and 94.94 % of the licking time in the early and late phases (*P < 0.01*). Aspirin significantly decreased the time of the late phase (70.39 %, *P < 0.01*) but did not markedly decrease the time of the early phase*.*

### Ammonia-induced coughing in mice

As shown in Table 1, EEM demonstrated marked anti-tussive activity. At 500, 250 and 125 mg/kg, EEM decreased the number of coughs induced by ammonia, with inhibition rates of 47.88, 34.18 (*P < 0.01*) and 14.30 % (*P < 0.05*), respectively, indicating a dose-dependent effect. Codeine (20 mg/kg) had an inhibition of 67.12 % (*P < 0.01*).

**Table 1:** Anti-tussive activity of the ethanol extract of M. punicea in the ammonia-induced coughs of mice test

Group	Dose (mg/kg)	Number of coughs	Inhibition (%)
Control	--	61.20 ± 6.88	--
EEM	500 i.p.	31.90 ± 7.81**	47.88
	250 i.p.	40.28 ± 8.64**	34.18
	125 i.p.	52.45 ± 7.13*	14.30
Codeine phosphate	20 i.p.	20.12 ± 4.56**	67.12

Each value represents the mean ± S.E.M. of 10 mice

*P < 0.05 compared with control. **P < 0.01 compared with control

### Sulfur dioxide-induced coughing in mice

As shown in Table 2, EEM demonstrated marked anti-tussive activity. Compared to the control group (107.71 ± 29.06), 500, 250 and 125 mg/kg significantly decreased the number of coughs induced by sulfur dioxide in a dose-dependent manner (15.71 ± 9.81, 30.14 ± 16.58 and 67.57 ± 19.26) (*P < 0.01*). Codeine (20 mg/kg) inhibited coughing by 81.82 % (*P < 0.01*).

**Table 2:** Anti-tussive activity of the ethanol extract of M. punicea in sulfur dioxide -induced coughing of mice

Group	Dose (mg/kg)	Number of coughs	Inhibition (%)
Control	--	107.71 ± 29.06	--
EEM	500 i.p.	15.71 ± 9.81**	85.41
	250 i.p.	30.14 ± 16.58**	72.02
	125 i.p.	67.57 ± 19.26	37.27
Codeine phosphate	20 i.p.	19.58 ± 7.82**	81.82

Each value represents the mean ± S.E.M. of 10 mice

**P < 0.01 compared with control

### Phenol red secretion in mouse tracheas

The results of the expectorant test are shown in Table 3. EEM decreased the excretive sputum in the trachea. Compared to 0.0733 absorbance in the control group, the absorbance value of three groups treated with EEM were 0.1608, 0.1051 and 0.0836, respectively. NH_4_Cl also showed good expectorant activity (*P < 0.01*).

**Table 3:** Anti-tussive activity of the ethanol extract of M. punicea in the phenol red secretion of mice tracheas

Group	Dose (mg/kg)	Absorbance (A)	Increase (%)
Control	--	0.0733 ± 0.019	--
EEM	500 i.p.	0.1608 ± 0.0301**	119.37
	250 i.p.	0.1051 ± 0.0246*	43.38
	125 i.p.	0.0836 ± 0.0178	13.32
NH_4_Cl	1500 i.p.	0.1451 ± 0.0147*	97.95

Each value represents the mean ± S.E.M. of 10 mice

*P < 0.05 compared with control.**P < 0.01 compared with control

### Acute toxicity

In the acute toxicity test, administration of EEM (500–2000 mg/kg) to mice did not cause death or acute behavior changes during the observation periods, and we also did not notice any pathology changes in mice. The LD_50_ was estimated to more than 2000 mg/kg, i.p. EEM was safe at the given dose in mice.

## Discussion

As an important folk medicine, *Meconopsis* plays an important role in traditional Tibetan (veterinary) medicines. Most plants of this genus are used to clear heat-evil and expel superficial evils, relieve coughing and asthma, eliminate inflammation and relieve pain for a long period of time. *M. quintuplinervia* and some species of the genus have been reported to exhibit analgesic and sedative activities [6]. Until now, the bioactivities of *M. punicea* have not studied. In this paper, based on its traditional use in treating pain, inflammation and coughing, the antinociceptive and antitussive activities of the ethanol extract of *M. punicea* flower were investigated. In the first phase of testing, the results showed that EEM has a significant analgesic activity and decreased the acetic acid-induced writhing response of mice and prolonged the latency time in hot plate test and the sleeping time in the barbiturate-induced sleeping time test. Meanwhile, it markedly decreased the time of licking in both the early and late phase in the formalin test. In the second phase of testing, the results showed that EEM had good anti-tussive activity. It decreased the number of coughs induced by ammonia and sulfur dioxide and the excretive sputum in the tracheas in a dose-dependent manner.

As a classical non-selective antinociceptive model, acetic acid produces a painful reaction and acute inflammation in the peritoneal area. It indirectly induced the release of endogenous mediators and stimulated nociceptive neurons [20, 21]. The results showed that EEM significantly inhibited the acetic acid-induced writhing response in a dose-dependent manner and presented a good peripheral analgesic effect. Then, the hot-plate test was used to evaluate the analgesic activity on the central nervous system [22], and the results indicated that EEM (500 mg/kg) markedly prolonged the latency time of mice and showed good central analgesic activity in the hot plate test. In the acetic acid-induced writhing response test, we found that the mice were sedated and slow after administrating EEM. The barbiturate-induced sleeping time test was finally carried out to evaluate the sedative activity of EEM. The results demonstrated that EEM markedly decreased the latency time of sleep and prolonged sleeping time of mice and had strong sedative activity.

Meanwhile, in order to validate the marked analgesic activity of EEM further, the formalin test was carried out. The test could be separated into two different phases in time; the first one was generated in the periphery through the activation of nociceptive neurons by the direct action of formalin, and the second phase occurred through the activation of the ventral horn neurons at the spinal cord level. Thus, it could be discriminated between central and peripheral pain components [16, 23]. Centrally acting drugs, such as opioids, inhibited both phases equally, while peripherally acting drugs, such as aspirin and aspirin only, inhibited the late phase [24, 25]. The results showed that EEM had a marked antinociceptive effect both the early and the late phase, while aspirin suppressed the later phase only (Fig. 6). From the results of the above tests, we suggest that the extract may act both centrally and peripherally to reduce pain.

Anti-tussive animal models were established by a mechanical, electrical and chemical stimulus. In our study, the anti-tussive activity of EEM was evaluated by ammonia-induced and sulfur dioxide-induced coughing in mice. These methods using a chemical stimulus were applied due to the simple procedure that omitted anesthetization; they are frequently used in new drug development [26]. From Table 1 and 2, EEM decreased the number of coughs induced by ammonia and sulfur dioxide. Meanwhile, because most expectorant drugs can increase secretion and dilute the sputum in the respiratory tract so that it could be expectorated easily with ciliary movement [17], the expectorant phenol red secretion in mouse tracheas was tested. As shown in Table 3, EEM markedly enhanced the tracheal phenol red output in a dose-dependent manner. These results were in accordance with the folk clinical uses of *M. punicea* to treat coughs and lung diseases, and the mechanisms should be studied further.

## Conclusion

The present study demonstrated that the ethanol extract of *M. punicea* flowers had good antinociceptive and anti-tussive activities *in vivo*. Further studies should be performed to investigate the mechanism of action and the active components of *M. punicea.*
